# Analysis of health concerns not addressed by REACH for low tonnage chemicals and opportunities for new approach methodology

**DOI:** 10.1007/s00204-023-03601-5

**Published:** 2023-09-27

**Authors:** Philip Botham, Mark T. D. Cronin, Richard Currie, John Doe, Dorothee Funk-Weyer, Timothy W. Gant, Marcel Leist, Sue Marty, Bennard van Ravenzwaay, Carl Westmoreland

**Affiliations:** 1 Syngenta Product Safety, Jealott’s Hill International Research Centre, Bracknell, RG42 6EY Berkshire UK; 2 School of Pharmacy and Biomolecular Sciences, Liverpool John Moores University, Byrom Street, Liverpool, L3 3AF UK; 3 BASF SE, Experimental Toxicology and Ecology, Carl-Bosch-Strasse 38, 67056 Ludwigshafen am Rhein, Germany; 4 Toxicology Department, Centre for Radiation, Chemical and Environmental Hazards, UK Health Security Agency, Harwell Science Campus, Chilton, OX11 0RQ UK; 5 School of Public Health, Imperial College London, London, UK; 6 Department of Biology and CAAT-Europe, University of Konstanz, 78457 Constance, Germany; 7 The Dow Chemical Company, Toxicology and Environmental Research and Consulting, 1803 Building, Midland, MI 48674 USA; 8 Department of Agrotechnology and Food Sciences, Wageningen University and Research, 6708 WG Wageningen, The Netherlands; 9 SEAC, Unilever, Colworth Science Park, Sharnbrook, MK44 1LQ Bedfordshire UK

**Keywords:** REACH, low tonnage chemicals, New Approach Methodologies, Risk Assessment

## Abstract

In Registration, Evaluation, Authorisation and Restriction of Chemicals (REACH) the criterion for deciding the studies that must be performed is the annual tonnage of the chemical manufactured or imported into the EU. The annual tonnage may be considered as a surrogate for levels of human exposure but this does not take into account the physico-chemical properties and use patterns that determine exposure. Chemicals are classified using data from REACH under areas of health concern covering effects on the skin and eye; sensitisation; acute, repeated and prolonged systemic exposure; effects on genetic material; carcinogenicity; and reproduction and development. We analysed the mandated study lists under REACH for each annual tonnage band in terms of the information they provide on each of the areas of health concern. Using the European Chemicals Agency (ECHA) REACH Registration data base of over 20,000 registered substances, we found that only 19% of registered substances have datasets on all areas of health concern. Information limited to acute exposure, sensitisation and genotoxicity was found for 62%. The analysis highlighted the shortfall of information mandated for substances in the lower tonnage bands. Deploying New Approach Methodologies (NAMs) at this lower tonnage band to assess health concerns which are currently not covered by REACH, such as repeat and extended exposure and carcinogenicity, would provide additional information and would be a way for registrants and regulators to gain experience in the use of NAMs. There are currently projects in Europe aiming to develop NAM-based assessment frameworks and they could find their first use in assessing low tonnage chemicals once confidence has been gained by their evaluation with data rich chemicals.

## Introduction

Over the last 80 years there has been growing understanding of the potential for chemicals to cause adverse health effects—their toxicity. Originally this was focused on severe effects following accidental poisoning with single high doses but as knowledge increased the range of potential adverse effects widened to include effects which might be seen following long-term exposure or to exposure during sensitive periods of development. An ever-growing list of experimental studies has been developed since the 1970s to assess the effects of chemicals and these studies have become incorporated into regulations on how the safety of chemicals should be assessed. Most of the studies use laboratory animals given high doses of chemicals to maximise the probability of detecting potential adverse effects. In many cases, the regulations take the form of lists of studies that must be done when certain triggers are met. In the case of the Registration, Evaluation, Authorisation and Restriction of Chemicals in the European Union (EU) regulation (REACH; ECHA 2022), the triggers are based on the annual tonnage produced or imported into the EU; the higher the tonnage, the more studies that must be done. The tonnage trigger is a pragmatic but clearly imperfect surrogate for potential human exposure, based on the premise that the higher the tonnage the greater the probability of more people being exposed to higher amounts of chemical. It also ties in with the economic reality that low tonnage products are unlikely to be able to bear the costs of extensive testing programmes. The studies required for each annual tonnage band in REACH are shown in Table 1.

**Table 1:** REACH study requirements for each tonnage band (TG represents an OECD Test Guideline)

Study requirements	< 1 tpa	1–10 tpa Annex VII	10–100 tpa Annex VIII	100–1000 tpa Annex IX	> 1000 tpa Annex X
In vitro skin irritation/corrosion TG 431, TG 439	−	+	+	+	+
In vitro eye irritation TG 467	−	+	+	+	+
In vitro skin sensitisation TG 442E	−	+	+	+	+
In vitro gene mutation in bacteria TG 471	−	+	+	+	+
In vivo acute toxicity: oral TG 401	−	+	+	+	+
In vitro mutagenicity study in mammalian cells or in vitro micronucleus study TG 487, TG 490	−	−	+	+	+
In vitro gene mutation in mammalian cells TG 476	−	−	+	+	+
In vivo skin irritation^a^ TG 404	−	−	+	+	+
In vivo eye irritation^a^ TG 405	−	−	+	+	+
Testing proposal for in vivo genotoxicity (if one of the in vitro tests is positive)	−	−	+	+	+
In vivo acute toxicity: inhalation TG 403	−	−	+	+	+
In vivo short-term repeated dose toxicity (28-day) TG 407	−	−	+	+	+
In vivo screening for reproductive/developmental toxicity TG 421, TG 422	−	−	+	+	+
In vivo sub-chronic toxicity (90 days) TG 408	−	−	−	+	+
In vivo pre-natal developmental toxicity in one species TG 414	−	−	−	+	+
In vivo extended one-generation reproductive toxicity (if triggered) TG 443	−	−	−	+	+
In vivo long-term repeated dose toxicity (≥ 12 months) (if triggered) TG 453	−	−	−	−	+
In vivo developmental toxicity in a second species TG 414	−	−	−	−	+
In vivo extended one-generation reproductive toxicity TG 443	−	−	−	−	+
In vivo carcinogenicity if triggered TG 451, TG 453	−	−	−	−	+

^a^In vivo study is only permitted if classification of the substance is not possible based on the in vitro results

## Health concerns to be addressed

In the process of mandating a list of studies for each tonnage band, it is easy to lose sight of the origins of the mandated studies as, in reality, being designed to address specific potential health concerns. These health concerns can be expressed in a series of questions:

- What are the effects if the chemical gets into someone’s eyes or on their skin?
- Can the chemical cause allergic dermatitis/respiratory sensitisation?
- What are the systemic effects of short-term exposure?
- What are the systemic effects of repeated exposure for a period of months?
- What are the systemic effects of exposure for many years?
- Can the chemical cause damage to genetic material?
- Can the chemical cause cancer?
- Can the chemical cause effects on fertility, the unborn child or the developing child?

The concerns relating to systemic effects reflect the three exposure durations identified by Ball et al. (2022) in their proposal to categorise exposures: short term (single or 1-day exposure), intermediate (repeat exposure for months), and extended (repeat exposure for many years). The health concerns are mirrored in the classification system (CLP) used in the EU (ECHA 2017), which is based on the Globally Harmonised System (GHS) system for classification and labelling (United Nations 2019). Both the REACH and GHS information requirements are based primarily on identifying potential hazards associated with a chemical. However, for this information to be of use in safety decision-making, the doses associated with causing these hazards, and the doses without effect, must be used in the context of the levels of the chemicals to which humans are actually exposed (locally and systemically). Thus classification schemes which do not take into account dose levels provide limited information (Doe et al. 2021).

## Analysis of health concerns addressed at each REACH tonnage band

Table 2 shows the health concern which is addressed by each study that is required in one or more of the REACH tonnage bands. An alternative way to look at each of the tonnage bands is to consider what health concerns are being addressed by the list of mandated studies for each band and, importantly, which health concerns are not addressed. The results of this analysis are shown in Table 3. The end result of an assessment in REACH is to provide a classification, health-based guidance values (HBGV—in the REACH regulation the HBGV is called a Derived No-Effect Level [DNEL]), and an exposure assessment (required for substances at > 10 tpa and fulfilling certain hazard categories or assessed as persistent). Consideration of the relevant list of mandated studies allows the status of each health concern for each tonnage band to be placed into one of three categories:

- **None**: no information is available to address the human health concern.
- **Some**: enough information on the health concern is available to identify substances with clear activity or no activity, but leaving some substances without definitive classification and/or DNELs/HBGVs for use in risk assessment.
- **Full**: sufficient information on the health concern to allow definitive classification according to CLP/GHS criteria and/or provide HBGVs for use in risk assessment that are considered to be broadly protective.

**Table 2:** The health concerns addressed by studies required by REACH (+ indicates the study is required, ± indicates it may be required)

Studies	Gene damage	Short term exposure	Skin/eye damage	Sensitisation	Inter-mediate exposure	Extended exposure	Cancer	Developmental and reproductive
In vitro skin irritation/corrosion TG 431, TG 439			+					
In vitro eye irritation TG 467			+					
In vitro skin sensitisation TG 442E				+				
In vitro gene mutation in bacteria TG 471	+						+	
In vivo acute toxicity: oral TG 401		+						
In vitro mutagenicity study in mammalian cells or in vitro micronucleus study TG 487, TG 490	+						+	
In vitro gene mutation in mammalian cells TG 476	+						+	
In vivo skin irritation^a^ TG 404			+					
In vivo eye irritation^a^ TG 405			+					
Testing proposal for in vivo genotoxicity (if one of the in vitro tests is positive)	+						+	
In vivo acute toxicity: inhalation TG 403		+						
In vivo short-term repeated dose toxicity (28-day) TG 407					+			
In vivo screening for reproductive/developmental toxicity TG 421, TG 422								+
In vivo sub-chronic toxicity (90 days) TG 408					+			±
In vivo pre-natal developmental toxicity in one species TG 414								+
In vivo extended one-generation reproductive toxicity (if triggered) TG 443					+			+
In vivo long-term repeated dose toxicity (≥ 12 months) (if triggered) TG 453						+		±
In vivo developmental toxicity in a second species TG 414								+
In vivo extended one-generation reproductive toxicity TG 443								+
In vivo carcinogenicity if triggered TG 451, TG 453							+	±

^a^In vivo study is only permitted if classification of the substance is not possible based on the in vitro results

**Table 3:**
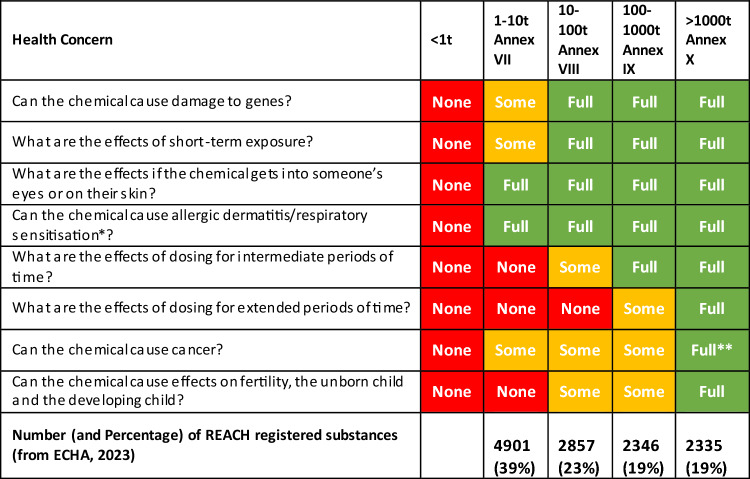
Analysis of information available for each REACH tonnage band

None: no information is available to address the human health concern. Some: enough information on the health concern is available to identify substances with clear activity or no activity, but leaving some substances without definitive classification and/or HBGVs for use in risk assessment. Full: sufficient information on the health concern to allow definitive classification according to CLP/GHS criteria and/or provide HBGVs for use in risk assessment that are considered to be broadly protective

*There is no specific assay for respiratory sensitisation, dermal sensitisation results are used to provide guidance

**Depends on whether chronic toxicity/carcinogenicity studies are triggered

There are no mandated studies in REACH for chemicals with annual tonnage of less than 1 tonne and, therefore, no evaluation can be made. In the 1–10 tonnage range, the focus appears to be on the effects following short-term exposure. It is possible to classify for skin and eye damage, and for dermal sensitisation. There is some evidence about the effect on genetic material, in vitro bacterial mutation which may trigger more studies, and some evidence on short-term exposure, from an acute oral study that has mortality as its primary endpoint. This study is unlikely to detect most non-lethal effects.

Some evidence about the effects of intermediate repeated exposure (28-day study) and the potential for effects on fertility and development (reproductive/developmental toxicity screening study which may trigger further studies) is gained in the 10–100 annual tonnage band. A DNEL/HBGV can be derived with additional uncertainty factors. These studies can also provide first evidence of effects on endocrine systems and potential carcinogenicity, but the level of confidence provided by these studies is not high enough for regulators to conclude absence of these effects, although genotoxic carcinogenicity can be excluded.

In the 100–1000 annual tonnage band, more evidence is added on intermediate exposure with a 90-day study to allow classification and derivation of DNEL/HBGV. Additional evidence on effects on development and fertility is added with a pre-natal developmental toxicity study in one species and a one-generation reproductive toxicity study (if triggered).

Over 1000 tonnes per annum, evidence can be provided on the effects of dosing for extended periods of time if a chronic toxicity/carcinogenicity study is triggered and on pre-natal development and fertility with an extended one-generation reproductive toxicity study and second species developmental toxicity. These studies allow classification and the derivation of DNELS/HBGVs for risk assessment. These studies can also confirm the presence or absence of an adverse effect on the endocrine-sensitive endpoints, but additional mode of action data would be required to confirm that an effect is mediated by an endocrine mode of action.

## Implications of the analysis: large number of substances with significant knowledge gaps

REACH demands a relatively meagre level of knowledge about many of the health concerns that may be associated with a chemical until annual tonnage is high. This appears to be based on the assumption that low tonnage chemicals need only to be assessed for health concerns associated with short-term exposure. Given the relatively low production volume, it is indeed unlikely that significant portions of the human population will be exposed to high quantities of low volume chemicals for an extended period of time. It could also be a consequence of the way toxicology studies are done in a sequential manner with the results of one study being used to set doses in the next study. It is a reality that there is increasing cost and complexity of the studies associated with providing evidence for longer term exposure and effects on development and reproductive toxicity.

Table 3 shows the number of substances registered in REACH at each tonnage band (data from ECHA 2023). Comparing the numbers and percentages with the number of health concerns with none, some or full evidence is illuminating; 39% of chemicals only have full evidence (as defined earlier) on skin and eye irritation and on dermal sensitisation health concerns; 81% do not have full evidence on extended exposure, cancer, or development and reproductive health concerns.

It would be important to investigate the physico-chemical properties and uses of the > 20,000 REACH registered substances to determine whether there is evidence on the health concerns which would be relevant for their use patterns. This might reveal that there are substances where there is prolonged exposure although production is in one of the lower tonnage categories, meaning these substances may not have sufficient data from REACH to be appropriately assessed. The opposite might also be true, there may be high tonnage substances where only short-term or very low levels of human exposure result from their use. It is inevitable that there are a large number of substances with mismatched exposure and knowledge.

## Implications of analysis: guidance for use of NAMs

The concept of addressing human health concerns rather than mandating a list of animal studies underlines the realisation that developing new approach methodologies (NAMs) on a study-by-study basis to replace specific animal studies will not be successful (Knight et al. 2021). Different lines of evidence from a variety of methods need to be drawn together. This is the rationale behind the development of Defined Approaches using a set of studies and interpretations and Integrated Approaches to Testing and Assessment (IATA) where information from different lines of evidence is brought together using weight of evidence approach. OECD has been developing this approach (OECD 2021). IATAs based on in silico and in vitro evaluations have been developed for skin (OECD 2017) and eye irritation (OECD 2019) and skin sensitisation (OECD 2016) where there is an established adverse outcome pathway (AOP) towards a specific health concern. There are also established frameworks for assessing mutagenicity (ECHA 2017). At the moment, schemes are addressing specific endpoints where it is possible to use specific lines of evidence to address stages in an AOP and contain fixed ways of integrating the evidence, although IATAs allow the different lines of evidence to be used.

The fixed approach raises two issues. Firstly, it is proving to be more difficult to address the broader health concerns such as intermediate and prolonged exposure, carcinogenicity and reproductive and developmental toxicity where many AOPs may operate. It will be difficult to base a scheme on a specific AOP or AOPs for broader health concerns, the current animal tests are designed to answer an open question (what effects can occur?) rather than a closed question (does a specific effect occur?). This is a major challenge for NAMs. It is likely that for these broader health concerns it will not be possible to create fixed processes for generating and evaluating lines of evidence as the evidence generation may be guided by chemical structure and the results of preliminary assays. Secondly, a scheme with a fixed set of tests is likely to become out of date as new methods are developed, which is a problem shared by traditional animal studies that have been mandated in legislation. Schemes should be able to evolve as new methods become available. The science associated with understanding human safety and the tools available to us are progressing with increasing speed. We must ensure that safety decisions can be made using the most up-to-date and relevant science and not get burdened with fixed lists of tests that may be out of date as soon as they are published. This will require ongoing review and replacement or supplementary processes where a new NAM test has advantages within a framework.

Legislation should specify the health concern to be addressed and not specify the methods. The types of methods suitable to address the health concerns depend on factors such as assay development and domain of applicability and thus, should be contained in guidance that can be revised as technology and knowledge improves to provide certainty of what is required to registrants.

An example of a different approach is the OECD Conceptual Framework for Testing and Assessment of Endocrine Disrupters (OECD 2018) which provides a structure into which lines of evidence on endocrine disruption can be placed and evaluated and into which new study types can be integrated as they are developed.

## Implications of the analysis: opportunity to use NAMs to improve assurance in low tonnage areas

The set of study requirements under REACH for > 1000 tonnage per annum chemicals covers the full range of health concerns. However, only 19% of chemicals, corresponding to 2335 chemicals, registered in REACH have this level of knowledge.

A similar observation has been made in the USA where it has been stated that less than 5% of the large number of chemicals currently in commercial use (approximately 50,000) have been fully tested (Fischetti 2010). This has highlighted the need for less resource intensive methods to be used and is a major driver in the USA where the US Environment Protection Agency (EPA) has devoted a large amount of resource to develop new technologies such as ToxCast (EPA 2022), which generates data and predictive models on thousands of chemicals of interest to the EPA.

It is a false assumption to conclude that 81% of chemicals registered in REACH have not been appropriately evaluated, but the tonnage driven study lists leave open the possibility that some of them may not have been. NAMs provide the opportunity for more knowledge about potential health effects to be gained for more chemicals and be related to their use and potential exposure.

The desire, by some, for the results of conventional animal studies to be predicted using NAMs has been a major stumbling block for their uptake and acceptance. The logic has been that the results of studies are needed particularly for classification and the development of DNELs/HBGVs. Thus, there is a movement towards looking for assessment methods that can place substances into an appropriate classification category and derive a protective DNEL/HBGV without necessarily having to predict the results of the animal studies that are currently used for this purpose. If we are constrained by trying to predict the outcome of animal studies exactly (that we know have their intrinsic biological and methodological variabilities), we will never transition to more relevant human health protection by being allowed to use human-based, rather than rodent-based science.

This approach opens the potential for assessment strategies that can identify the substances of the greatest concern and the substances of least concern in tonnage bands where there is currently no knowledge for several health concerns. 39% of substances registered in REACH, corresponding to 4901 chemicals, are in the 1–10 tonnage band and they have little to no information on: effects of dosing for intermediate periods; extended periods (including effects on the endocrine system); and effects on fertility, the unborn child and the developing child. They also only have limited knowledge on cancer causation (genotoxicity).

There are indications that NAMs are capable of providing some knowledge in these areas and so may allow identification of high levels of concern and low levels of concern (Ball et al. 2022; Baltazar et al. 2020; Dent et al. 2021; Fentem et al. 2021; Mahony et al. 2020; Middleton et al. 2022; Moné et al. 2020; Rajagopal et al. 2022). There is an opportunity to replace data gaps with knowledge for substances in the 1–10 tonnage band and at the same time develop and gain confidence in NAMs towards their eventual use in higher tonnage bands. The REACH legislation is clear that animal testing must be used only as a ‘last resort’ and that non-animal approaches should be used where possible. However, the criteria currently outlined within REACH for the use of NAMs:

1. results are derived from an in vitro method whose scientific validity has been established by a validation study, according to internationally agreed validation principles;
2. results are adequate for the purpose of classification and labelling and/or risk assessment; and
3. adequate and reliable documentation of the applied method is provided;

make it difficult to use them in higher tonnage bands, although it has been suggested that the criteria can be met with current NAMs (Ball et al. 2022), but there is no such restriction for the 1–10 tonnage band where the number of mandated studies is so limited. The NAMs are not replacements for traditional animal toxicology studies, but they provide additional knowledge that may otherwise not be gained. There are several projects (ECETOC 2023; Mansouri et al. 2021; RISK-HUNT3R 2023; Westmoreland et al. 2022) that are developing frameworks for the use of NAMs for this purpose using reference chemicals to provide the necessary level of confidence.

It should be recognised that this approach would provide data on health concerns where there is currently little or no information and, therefore, no classification has been made. The current lack of knowledge on chemicals in this tonnage band restricts their use but could still allow chemicals of concern to be used or conversely could limit the use of substances that could improve sustainability. Applying NAM-based assessment may subject these chemicals to restrictions they would not otherwise be subject to. It would be important to establish that the restrictions are justifiable and not excessively precautionary by appropriate calibration of the NAM-based assessment and to assure that chemicals considered to be of low concern can be used with confidence.

## Conclusions

The range of concerns over the possible adverse effects of chemicals has become extended over the last 50 years, starting in the 1970s with concerns over the effects of single high exposure on mortality, skin and eye irritation, and dermal sensitisation. There are now eight areas of health concern, with repeated and prolonged systemic exposure, the effect on genetic material, cancer, and fertility and development added to the initial acute concerns. Endocrine disruption has recently been added as a hazard category although it could be argued that the studies which have been used to cover the existing areas of health concern would also identify endocrine related effects; indeed to be identified as an endocrine disruptor there has to be an adverse effect in a conventional study that can be demonstrated to be caused by an endocrine mechanism (Andersson et al. 2018). Over time, study designs (primarily in animals) have been developed and agreed internationally (OECD 2009) to assess these areas of concern and these studies have become incorporated into regulations aimed at ensuring the safe use of chemicals. Within the EU in REACH, the criterion for deciding the studies that must be performed is the annual tonnage volume of the chemical which is manufactured or imported into the EU with the study requirements increasing with increasing tonnage band.

We have analysed the mandated study lists for each annual tonnage band in REACH in terms of the information they provide on the eight areas of health concern (Table 3). We have correlated this analysis with the number of substances registered in REACH for each tonnage band and it is clear that very few substances are likely to have complete datasets on all the areas of health concern covered by REACH (mandated to have full information on all the areas of concern (19%) and 62% are mandated to only have information on short-term exposure, sensitisation and effects on genetic material).

This analysis suggests opportunities for the adoption of NAMs into REACH to provide additional knowledge to assess the safety of chemicals. It underlines the trend in NAM development to move away from study-by-study replacement by recognizing that the conventional studies are designed to provide information on the relevant health concern. Strategies which use combinations of NAMs to address particular health concerns are being developed and these should be judged on whether they result in equivalent levels of protection in terms of classification category and HBGV rather than being able to predict the results of the conventional animal studies. Paul Friedman et al. (2023) conducted a review of the variability and relevance of existing mammalian toxicity tests, specifically when it comes to assessing impact on human health. The goal of this study is to set data-driven and science-based expectations for NAMs based on the variability and relevance of the traditional toxicity testing models. Qualitative reproducibility of organ-level effect observations in repeat dose studies in adult animals was 39–88% (Paul Friedman et al. 2023) so it is important that NAMs are not held to a higher standard. There is emerging evidence that classification and HBGVs can be achieved in several areas of health concern, such as repeat dose toxicity and cancer, using NAMs (Ball et al. 2022).

This analysis has quantified the concerns raised by Berggren and Worth (2023) over the paucity of information available for substances in the lower tonnage bands, especially the 1–10 annual tonnage band. Deploying NAMs for health concerns, such as repeat and extended exposure and carcinogenicity, would not be as replacements of existing studies but they would provide additional information relating to these health concerns. There would be value even if the NAMs were only capable of categorising substances at higher or lower levels of concern, although they have been shown to be able to provide HBGVs. This would be consistent with current European Commission considerations on whether changes in standard information requirements and Annex XI could be included in REACH revision to identify most harmful substances (Schutte 2023). As suggested by Berggren and Worth (2023), employing NAMs at this tonnage band would be a way for registrants and regulators to gain experience in their use and interpretation. It is important that NAM-based assessment is appropriately calibrated to provide appropriate assessments of safe.

## Data Availability

Data are not available, the purpose of this paper was to provide comment rather than data.
